# Injury Epidemiology in Elite U17 Football Players: A Prospective Study Across Six Competitive Seasons

**DOI:** 10.3390/life16040632

**Published:** 2026-04-09

**Authors:** Tomislav Pranjić, Frane Žuvela, Toni Modrić, Marko Stojanović, Ante Bandalović, Ante Turić, Tomislav Barić, Jakša Škomrlj, Šime Veršić

**Affiliations:** 1 Faculty of Kinesiology, University of Split, 21000 Split, Croatia; tomislav.pranjic@kifst.eu (T.P.); frane.zuvela@kifst.eu (F.Ž.); toni.modric@kifst.hr (T.M.); skomrljj@gmail.com (J.Š.); 2 HNK Hajduk Split, 21000 Split, Croatia; ante.bandalovic@hajduk.hr (A.B.); ante.turic@hajduk.hr (A.T.); tomislavbari782@gmail.com (T.B.); 3 Faculty of Sport and Physical Education, University of Novi Sad, 21000 Novi Sad, Serbia; marko.stojanovic@fsfvns.edu.rs; 4 Training Expertise Laboratory, 21000 Novi Sad, Serbia; 5 Department of Orthopedics and Traumatology, Surgery Clinic, University Hospital Split, 21000 Split, Croatia; 6 FC Noah, Yerevan 0023, Armenia

**Keywords:** football, injury, incidence, youth, under 17

## Abstract

Injuries in elite youth football may disrupt training continuity and long-term development, particularly during the post-peak height velocity (post-PHV) period when training and match demands increase. Therefore, the aim of this study was to determine injury incidence and describe injury patterns in elite U17 football players across six consecutive competitive seasons, including injury type, anatomical location, mechanism, recurrence, and severity. A prospective longitudinal injury-surveillance design was implemented in one elite football academy from 2016/2017 to 2021/2022. Injuries were recorded in the club’s medical database using the international consensus time-loss definition. Injury incidence per 1000 h was calculated for overall exposure, training, and matches, and injuries were analyzed by diagnosis, mechanism, recurrence, and severity. Across the study period, 331 injuries were recorded. Overall injury incidence was 6.95/1000 h, with markedly higher incidence in matches (20.61/1000 h) than training (5.82/1000 h). Seasonal incidence ranged from 4.49/1000 h in 2019/2020 to 9.31/1000 h in 2021/2022. The proportion of injured players ranged from 48% to 76% per season. The most frequent injuries were contusions and muscle cramps/DOMS, followed by ligament injuries, tendinosis, and muscle ruptures. Knee, thigh, ankle, and hip/groin were the most affected regions. Most diagnoses showed a predominantly non-contact pattern. These findings support targeted prevention and load-management strategies in elite youth football.

## 1. Introduction

Football is a complex sport characterized by frequent fluctuations in load, where periods of low and moderate intensity alternate with short but recurring bouts of high-intensity activity [1,2]. During a match, players repeatedly perform accelerations and decelerations, changes in direction, jumps, and kicks, as well as contacts and duels, with substantial mechanical stress placed on the lower-extremity muscle–tendon, ligamentous, and bone structures [1,3]. These demands require a high level of aerobic and anaerobic capacities, neuromuscular control, and tolerance to repeated eccentric stress [4,5]. In senior professional football, players typically cover 9–13 km per match, with approximately 10% of the total distance performed at high-speed running (HSR) intensity and 1–4% at sprinting distance (SPR) [6,7,8]. A similar relative contribution of HSR and SPR to total match distance has also been reported in elite youth football players [9,10]. Although these high-intensity activities account for a relatively small proportion of the total distance covered, they are particularly important because they may substantially influence situational efficiency, acute fatigue, and cumulative tissue stress, especially under conditions of fatigue and fixture congestion. [11].

Accordingly, professional football is a sport with a relatively high incidence of injuries [12]. Reported estimates vary across studies, ranging from 1.6 to 19.2 injuries per 1000 h of exposure [13,14,15]. In contrast, a systematic review and meta-analysis reported a pooled estimate of 8.1 injuries per 1000 h of exposure [16]. Because injuries at the professional level create financial and team performance-related challenges for clubs, interest in investing in and improving preventive programs within club systems has been increasing [17]. Consequently, at the professional male club level, overall injury incidence has declined over time by approximately 3% per season [18]. However, despite the reduction in overall injury burden, the proportion of muscle injuries, particularly hamstring injuries, has not decreased and has even increased in professional elite male football [19].

Injuries resulting in time loss and developmental stagnation are also common among younger football players and in academy settings [20]. Football academies aim to prepare players for the physical and tactical demands of senior football through systematic long-term development and continuous training [21]. Accordingly, long-term athlete development (LTAD) necessitates a progressive increase in training volume, intensity, and complexity, while taking into account biological maturation and individual growth rates [21]. As performance demands increase with age and biological processes such as peak height velocity (PHV) influence injury risk, injury epidemiology should be monitored across age categories [22]. The literature identifies PHV, typically corresponding to the U15 age group in boys, as a period of elevated injury risk, emphasizing the need to adapt training and preventive programmes to maturation status [22]. Furthermore, there is some evidence indicating that injury risk is also elevated in the period after PHV, which is attributed to a combination of maturational changes and increased physical demands [23,24]. Specifically, the transition into the U17 age group may represent a turning point, as many players are likely to have completed, or be approaching the end of, the PHV period and are entering a later phase of adolescence (post-PHV) [25]. During this period, clubs typically increase training and competition demands, which can potentially create a favorable environment for injury occurrence [26,27]. Precisely due to the completion of PHV and the simultaneous rise in volume and intensity, we can expect changes in injury epidemiology and injury profiles in post-PHV age groups [22]. Therefore, establishing injury epidemiology at the age when more intensive training programs begin, corresponding to the U17 age group, is of exceptional importance to develop optimal programs for injury reduction within football academies.

The injury profile of elite U17 football players has been investigated previously, and such data provide valuable information. In general, the overall injury incidence in elite U17 players is slightly above 8 injuries per 1000 h of exposure, with a higher incidence reported in matches than in training [23,28,29,30,31,32,33]. Most injuries affect the lower extremities, particularly the ankle, thigh, and knee, while ligament sprains and muscle injuries are among the most frequent diagnoses [20,32,34]. Injury mechanisms are often non-contact, and injury severity is usually mild to moderate, although severe injuries may still account for a meaningful proportion of cases [32,34]. However, reported injury incidence in elite U17 football players varies considerably, ranging from 1.9 to 19.4 injuries per 1000 h of exposure, likely due to differences in injury-surveillance methodology and sample size [35]. In addition, most studies have covered only 1 to 3 seasons and often included multiple age categories simultaneously [23,29,30,31,32]. Despite the existing body of research, long-term prospective injury surveillance conducted within a single elite academy remains scarce, limiting consistent and comparable insight into the injury epidemiology of U17 players [23,29,30,31,32,33]. Moreover, the present dataset spans the period of COVID-19 restrictions and the subsequent return to regular training and competition, which may have influenced exposure and injury risk [36].

Accordingly, this study aims to determine the incidence of injuries in elite U17 football players during a systematic training and competition process within a football academy, and to provide a detailed insight into the injury profile in terms of location, type, mechanism of occurrence, and severity of injuries in the U17 age group. An additional aim is to describe the distribution of injuries with respect to training and matches to identify potentially critical load points. The resulting data should enable a more realistic assessment of risk patterns in late adolescence and serve as a basis for improving specific preventive strategies within elite football academies, particularly in the U17 age category.

## 2. Materials and Methods

### 2.1. Study Design

This longitudinal study examined the incidence and characteristics of injuries in an elite football academy over six consecutive competitive seasons, spanning from the 2016/2017 season to the 2021/2022 season. Injury data were collected systematically throughout the entire observation period and were extracted from the academy’s internal medical database. The observation period encompassed the pre-season and competitive phases of each season, while the transition period, which lacked an organized training process, was excluded from the analysis. All players were informed about the purpose of the study, and, as the participants were youths, consent for the use of data was obtained from parents or legal guardians. The study was conducted in accordance with the ethical principles of the Declaration of Helsinki and was approved by the Ethics Committee of the Faculty of Kinesiology (Ethical Approval Number: 2181-205-02-05-23-0007).

### 2.2. Participants

The sample consisted of registered male U17 football players from an elite football academy in Split, Croatia, competing at the highest level of the national championship. From the 2016/2017 to 2021/2022 seasons, a total of 289 players were recorded. Because the study covered six consecutive seasons, player turnover between seasons was expected. Therefore, the analyses were performed on a season-by-season basis, with players included only in the seasons in which they were registered and actively participated in the training and competition process. Across the six seasons, a total of 331 injuries were recorded. Due to regular player turnover during the winter transfer window and to avoid bias in the results, seasonal analyses included only players who actively participated in the training and competition process during the respective season. During the season, players participated in a regular training process that included multiple training sessions per week and one official match per week, with at least one day off from organized football activities.

### 2.3. Variables

Injuries were recorded by the club’s medical staff during routine daily examinations immediately before or after training sessions and matches. Training sessions and home matches were predominantly performed on artificial turf. All data were collected and classified in accordance with the consensus statement on injury definitions and data collection procedures in football injury research [37]. Injuries were classified by type as contusions, muscle cramps (DOMS), tendinosis, ligament injury (dislocations/sprains), muscle ruptures, other bone injuries, fractures, meniscus or cartilage lesions, bursitis, tendon ruptures, concussions, abrasions, and lacerations. In the present study, muscle cramps/DOMS were classified as a separate diagnostic category and were included in the analysis only when they led to time loss or to modification/interruption of training or match participation, in line with the applied time-loss injury definition. For each injury, the number of days absent from full training and competition was recorded, along with the circumstances of occurrence (training or match), mechanism of injury (contact or non-contact), and recurrence. Re-injury was defined in accordance with the consensus guidelines as an injury to the same anatomical region and of the same type occurring after the player’s return to full participation in training and competition. Injury severity was classified based on days of absence, while in the analysis, it was presented as the total number of days absent (days out). Injury distribution by affected body region was also analyzed, as well as the overall injury burden, defined as the product of the number of injuries and the number of days absent. Football exposure was calculated for all observed seasons and expressed in terms of hours of training and match play. Total match exposure time was calculated as the product of the number of team matches played, the number of players in the team, and match duration in minutes, divided by 60, while total training exposure time was calculated as the product of the number of players present in training and training duration in minutes, divided by 60. Depending on the season and competition calendar, the team typically played approximately 26–32 official matches per season. The pre-season period lasted approximately 6 weeks, while the off-season transition period lasted around 4 weeks and did not include organized team activities. Injury incidence was calculated as the number of injuries per 1000 h of exposure, separately for training, matches, and the overall observation period. Additionally, the seasonal incidence rate of injured players was calculated and expressed as the percentage of injured players to the total number of registered players per season.

### 2.4. Statistics

Data were analyzed using descriptive statistics. Results are presented as arithmetic means and relative frequencies. Injury incidence is expressed as the number of injuries per 1000 h of exposure with corresponding 95% confidence intervals. In addition, injury incidence was calculated separately according to overall exposure, training exposure, and match exposure, and was stratified by season and injury type to provide a more detailed description of injury occurrence, severity, and burden. The analysis included comparisons of incidence between seasons and between training and matches. Statistica v.14.0.1.25 (TIBCO Software Inc., San Ramon, CA, USA), Microsoft Excel 2019 (Microsoft, Redmond, WA, USA), GraphPad Prism v.10.6.1 (GraphPad Software, Boston, MA, USA) and MedCalc Statistical Software v.23.4 (MedCalc Softwate Ltd., Ostend, Belgium) were used for the analysis. The level of statistical significance was set at *p* < 0.05.

## 3. Results

Table 1 shows the injury incidence rates across 6 seasons. Overall incidence ranged from 4.49 (95%CI: 4.45–4.55) in 19/20 to 9.31 (95%CI: 9.24–9.38) in 21/22. Match incidence was consistently higher than training incidence in every season, peaking at 29.51 (95%CI: 29.08–29.94) in 21/22, while the lowest match incidence was observed in 18/19 (9.84 (95%CI: 9.59–10.09)).

A total of 331 injuries were recorded across the observed period, with the seasonal proportion of injured players ranging from 48% (19/20) to 76% (2016/2017) (Table 2). The highest number of total injuries occurred in 2021/2022 (N = 70), while the lowest was recorded in 2019/2020 (N = 32).

The most frequent injury types were contusions (N = 62) and muscle cramps (DOMS) (N = 62), followed by ligament injury (N = 57) tendinosis (N = 49) and muscle rupture (N = 41) (Table 3). The greatest average time-loss was observed for meniscus/cartilage lesions (109.4 days), while other high-burden injuries included tendon rupture (63.7 days) and fracture (63.6 days) and tendinosis (48.9 days). Reinjury counts were most pronounced for ligament injury (N = 13) and muscle cramps (DOMS) (N = 10), and most injury types occurred more often in training than in matches, with several categories showing a predominantly non-contact pattern (e.g., muscle cramps (DOMS) and tendinosis).

**Figure 1:**
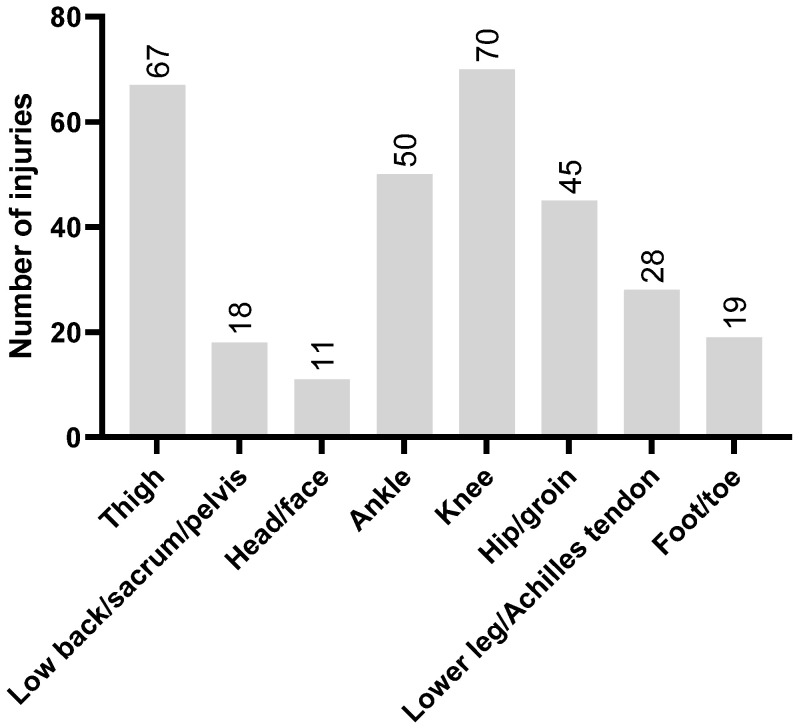
Injured body parts.

Figure 1 indicates that injuries were most located at the knee (N = 70) and thigh (N = 67), followed by the ankle (N = 50) and hip/groin (N = 45). Lower frequencies were observed for the lower leg/Achilles tendon (N = 28), foot/toe (N = 19), low back/sacrum/pelvis (N = 18), and head/face (N = 11).

**Figure 2:**
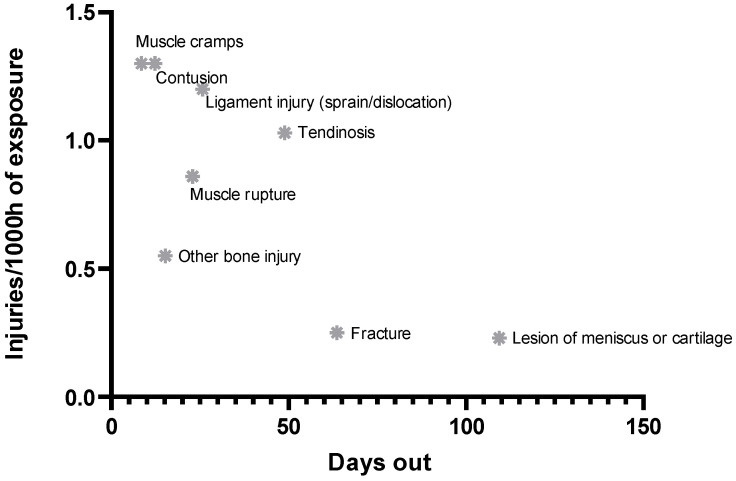
Injury burden.

Figure 2 presents an incidence–severity profile of the recorded injury types by combining injury incidence (injuries/1000 h of exposure) with time-loss (days out). Injuries located in the upper-left part of the figure, such as muscle cramps (DOMS) and contusions, were relatively frequent but generally associated with shorter absences, indicating a burden driven mainly by high occurrence. In contrast, tendinosis combined a substantial incidence with longer time-loss, suggesting a more pronounced overall burden. Meniscus/cartilage lesions and fractures were less frequent but resulted in the longest absences, indicating a burden primarily driven by injury severity rather than incidence.

**Figure 3:**
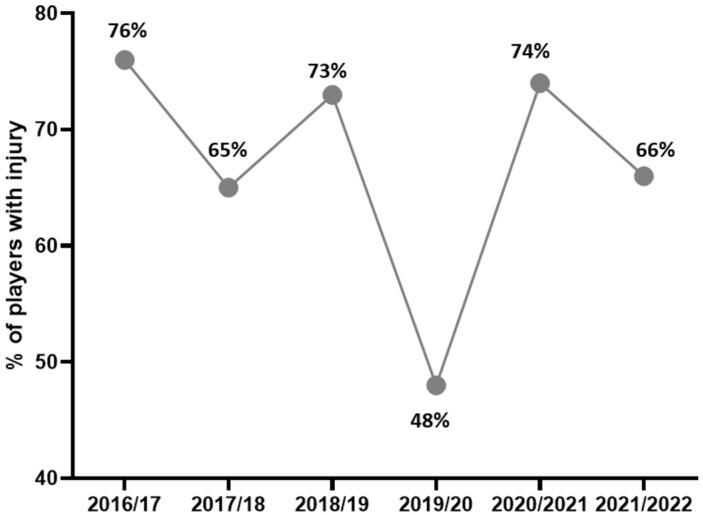
Proportion of injured players.

Figure 3 shows that the seasonal proportion of injured players fluctuated across the study period. The lowest value was observed in 2019/20 (48%), followed by a sharp rise in 2020/2021 (74%), with values remaining high in the surrounding seasons (range 65–76%).

**Figure 4:**
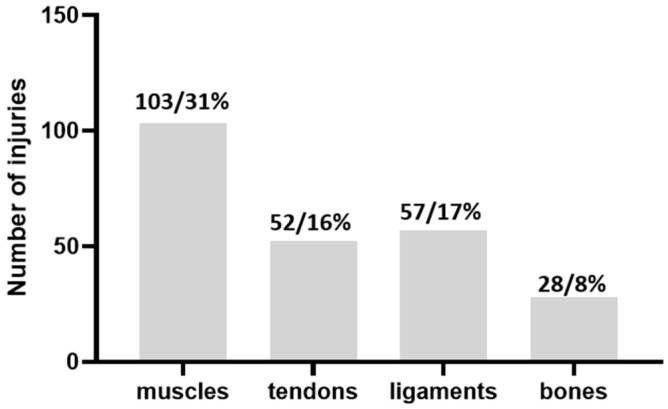
Injuries per body tissue (N/percentage of total injuries).

Figure 4 presents the distribution of injuries by affected tissue, with muscle injuries (N = 103; 31%) being the most frequent category. This was followed by ligament injuries (N = 57; 17%), tendon injuries (N = 52; 16%), and bone injuries (N = 28; 8%). The remaining injuries were attributed to other/unspecified tissue categories, which are not shown in the figure.

## 4. Discussion

This study aimed to describe the epidemiology of injuries in elite U17 football players over six consecutive seasons in terms of injury incidence, type, location, and severity. Accordingly, several main findings emerged. Specifically, the overall injury incidence varied across the observation period, with higher rates at the end of the period compared to the beginning. A higher incidence was recorded during matches than during training, with a large proportion of players sustaining an injury during the season. Furthermore, acute injuries were the most common, and they were generally minor, such as contusions and muscle-related problems. Finally, injuries predominantly affected the lower extremities, most often the knee and thigh, and regarding the injured tissue, muscle injuries were the most prevalent.

### 4.1. Injury Incidence

Overall, across the six-season period, injury incidence was consistently higher in matches than in training, confirming the greater risk associated with competitive exposure. A review of the literature indicates fluctuations in annual injury incidence among players of this age group [35,38,39]. Specifically, a study of 223 elite players reported an overall injury incidence of 8.28 per 1000 h for the U17 age group, with 7.24 per 1000 h in training and 16.77 per 1000 h in matches [38]. In this context, our findings suggest the increased risk during competitive exposure, likely due to the greater physical and psychological demands [40]. Conversely, a study on elite German U17 players conducted over a single competitive season reported a total injury incidence of 2.5 per 1000 h, with 1.8 per 1000 h in training and 0.9 per 1000 h in matches, which is substantially lower than our findings [39]. These differences may be attributable to methodological variations between studies. Notably, our study encompasses six consecutive seasons of monitoring within the same system, which should minimize the influence of single-season deviations and methodological variability, providing a more stable and consistent estimate.

Furthermore, a comparison between the first season (2016/2017) and the sixth season (2021/2022) showed that the injury incidence was lower in the first than in the final observed season (IRR = 0.78; 95% CI 0.77 to 0.79). This finding indicates that a continuous reduction in injury incidence was not achieved over the observed period, despite the systematic implementation of prevention programs within the academy. The prevention programmes included continuous strength and power training, coordination and balance training, as well as standardized warm-up protocols based on FIFA 11+ program, whose positive effects on injury reduction and selected performance-related outcomes have been previously documented in the literature [41]. The protocols were supervised by the authors of this study, who also served as coaches within the observed academy. However, although we did not measure directly, this trend can be explained by several external factors. First of all, the 2019/20 season was marked by the COVID-19 pandemic. In that season, the lowest overall incidence was recorded (4.49 per 1000 h), which can be explained by reduced exposure, meaning a lower density of training and especially competition, and by the fact that the interruption occurred in the part of the season when a higher number of injuries is usually expected due to accumulated fatigue [42,43]. In other words, the lower incidence in that season does not necessarily reflect a true reduction in risk, but primarily reduces exposure to load, particularly in the context of a reduced number of matches. Moreover, our results show a pronounced increase in injury incidence in the post pandemic seasons. This trend has already been documented in some studies in elite senior football players [44,45]. It is plausible that a similar pattern can also be expected in younger age groups. A comparable pattern was observed in a longitudinal analysis of elite youth football players, in which the pre-pandemic downward trend in injury incidence was disrupted during the COVID 19 period, followed by an increase in incidence in the pandemic season and the immediately post pandemic season [36]. The congested competition schedule after the season restart increased the cumulative load within a short time frame. The synergistic effect of reduced preparedness and a sudden rise in load is likely a key mechanism explaining the increased incidence in the period after COVID. Additionally, as the mechanisms underlying the observed trend were not directly assessed in this study, any interpretation regarding factors associated with the higher incidence remains speculative. It is possible that prevention programmes do not reach their full effectiveness without long-term and continuous implementation, since their protective effect may diminish when the training process and competitive engagement are disrupted. Consistent with this assumption, some studies suggest that five weeks without normal training can lead to significant declines in speed, strength, and aerobic capacity in elite athletes [46]. Furthermore, academy-specific contextual factors, including the frequent use of artificial turf as the main training surface and coaching changes during the observation period, may also have played a role [47,48]. However, as their influence was not directly assessed in the this study, these considerations remain speculative.

In parallel with the overall injury incidence, the proportion of injured players in our study ranged from 48% to 76%, with the highest value recorded in the 2016/2017 season at 76%. This pattern is comparable to findings reported in other elite youth academy settings [32]. In this context, our results support the view that injuries affect most players within a single competitive season in elite U17 football. From a practical perspective, this means that in a typical elite U17 squad of 25 players, approximately 19 players can be expected to sustain at least one injury leading to absence from the training process during the season.

### 4.2. Injury Classification by Type and Tissue

The analysis by injury type showed that the most common injuries in our sample were contusions (N = 62) and muscle cramps (DOMS) (N = 62), followed by ligament injury (N = 57), tendinosis (N = 49), and muscle ruptures (N = 41). However, the distribution by frequency does not fully reflect their true burden. Although contusions and muscle cramps (DOMS) accounted for the largest number of injuries, their average time loss was relatively short, at 8.5 and 12.3 days, respectively, indicating a smaller individual impact on training continuity. In contrast, less frequent injuries, such as meniscal or cartilage lesions (109.4 days), tendon ruptures (63.7 days), fractures (63.6 days), and tendinosis (48.9 days), resulted in a substantially greater number of days lost. This shows that injury burden is not determined by incidence alone, but by the combination of incidence and injury severity, whereby a smaller number of more severe injuries can have a greater overall impact than many minor injuries.

When injuries were classified according to the affected tissue, muscle injuries accounted for 31% of all injuries (N = 103), ligament injuries for 17% (N = 57), tendon injuries for 16% (N = 52), while bone injuries accounted for 8% (N = 28) of the total. Together, muscle and tendon structures comprise almost half of all injuries in our sample, which further confirms the dominant load placed on the musculotendinous system in this age group.

A comparison with previous studies shows a similar overall pattern in the distribution of injury types. In a ten-season follow-up of an elite French academy, contusions were the most common, at approximately 30%, followed by muscle strains at approximately 15 to 17% and ligament sprains at approximately 16% [40]. In a four-season study of an elite national academy in the U17 category, muscle injuries accounted for 22% of all injuries, superficial contusions for 17%, and joint sprains for 13% [32]. Furthermore, in a three-year prospective follow-up of elite U17 players, muscle injuries accounted for 61.4% of all injuries, while contusions accounted for 20.5% [33].

Our results are largely comparable to these findings, since contusions were also among the most common individual diagnoses in our sample [32,49]. In addition, when muscle cramps, muscle ruptures, and tendinopathies are considered together, musculotendinous injuries account for the largest share of the total number of injuries in our sample, which is consistent with the dominant proportion of muscle injuries in the U17 population described in the literature [32,33].

Despite these differences in percentages, the overall injury profile in our study aligns with the patterns described in previous research on elite U16 to U17 football, where muscle and soft tissue injuries predominate, while ligament and bone injuries constitute a smaller but stable proportion of the overall injury structure.

### 4.3. Injuries by Body Parts

In our sample, injuries predominantly affected the lower extremities, with the most common knee injuries (N = 70, approximately 21.1%) and thigh injuries (N = 67, approximately 20.2%), followed by ankle (N = 50, approximately 15.1%) and the hip and groin region (N = 45, approximately 13.6%).

Comparison with previous research shows a similar set of predominantly injured regions, although the ranking varies depending on the context and the monitoring methodology [32,33,38,50,51]. In a prospective follow-up of elite U17 players in a national team programs, the largest proportion of injuries occurred in the thigh (31.8%) and the hip and groin region (25%), whereas the ankle (9.1%) and especially the knee (6.8%) were less frequent [33]. In our sample, the thigh, hip, and groin region were also among the leading locations, but the knee was more prominent and represented the most frequently injured region. This difference may be related to different exposure patterns (matches and trainings) in club versus national team settings, also as the different playing style between teams and national teams. It may also reflect differences in injury documentation and grouping methodology. Similarly to our findings, in an English elite academy across four seasons, the most common injury locations were the thigh (23%), knee (17%), ankle (17%), and hip and groin region (9%), which is quite similar to our findings [50]. Additional context is provided by a Danish study of elite U17 players, which found that hip and groin injuries had the highest incidence, while knee injuries had the greatest burden, expressed as days lost per 1000 h [38].

The reported injury locations in our study, as well as in previous studies, are not surprising given the nature of football. The lower extremities bear the greatest game-specific mechanical stress. Repeated sprinting, frequent accelerations and decelerations, frequent changes in direction, and landing tasks generate high forces at the knee and ankle, while kicks, sliding tackles, and contact situations further increase the risk of injury [52]. It is also possible that some of the observed injury patterns are influenced by physical changes occurring during adolescence [22,53]. Rapid growth, temporary alterations in neuromuscular control, and changes in the relationship between body size, strength, and coordination may contribute to the susceptibility of certain anatomical regions to injury during this developmental period [22]. In addition, repeated unilateral patterns, such as passing and shooting with the dominant leg, and high cumulative exposure across training and matches place a particular load on the hip and groin complex, which is why these regions emerge as critical points in football [54]. Therefore, the quality of physical training programmes are important in young football players, and maturational status should be monitored to ensure that training loads and preventive content are appropriately adjusted to the developmental stage of each player.

### 4.4. Strengths and Limitations

This study has several limitations that should be considered when interpreting the results. First, the sample included players from a single elite football academy, which may limit the generalizability of the findings to other systems, levels of competition, or geographical contexts. However, focusing on one elite academy provides a relatively homogeneous environment with structured training and a medical framework, allowing for consistent injury monitoring across multiple seasons. At the same time, this single-academy design may limit the external validity of the findings, since training methodologies, medical practices, and competition structures may differ across academies. In addition, despite a clear medical protocol for injury diagnosis and treatment, it is possible that a small number of injuries remained undocumented due to players underreporting symptoms or continuing to train despite complaints, which may have led to an underestimation of the true injury incidence.

Despite these limitations, the study has several important strengths. First, it involved longitudinal monitoring across six consecutive seasons within the same system, which reduces the influence of short-term seasonal variations and enables a more stable assessment of injury epidemiology. Moreover, data were collected systematically and classified in accordance with the international consensus on the definition and reporting of injuries in football, ensuring methodological consistency and a high level of data reliability. A particular strength of the study is the detailed analysis of injuries by type, location, affected tissue, mechanism of injury, and time loss, which provides deeper insight into the true injury burden within an elite U17 population. The combination of long-term monitoring and detailed classification, therefore, makes this study a valuable contribution to the understanding of injuries in this age group.

## 5. Conclusions

This study, conducted over six consecutive seasons at an elite U17 academy, found that overall injury incidence was 6.95 (95% CI 6.92 to 6.97) injuries per 1000 h of exposure, with match incidence consistently higher than training incidence, and that a large proportion of players sustained at least one injury within a season. The most common diagnoses were contusions and muscle cramps (DOMS). Injuries predominantly affected the lower extremities, particularly the knee and thigh, followed by the ankle and the hip and groin region. Regarding affected tissue, muscle injuries accounted for the largest share of all injuries, followed by ligament and tendon injuries, while bone injuries represented the smallest proportion.

Since injuries most commonly involve the knee, thigh, ankle, and hip, prevention in U17 players should prioritize these regions. Alongside general preventive approaches, such as strength and power training, balance training, and structured warm-up protocols, short micro-programs targeting more vulnerable regions could also be considered. Exercises targeting knee stability, eccentric thigh-muscle strength, and ankle proprioception may be relevant. Preventive content may also benefit from individualization according to injury history and identified player-specific deficits. Future research should include multiple academies, other age groups, different regions, both genders, and players of different competitive levels to provide a broader understanding of injury epidemiology in youth football. Also, the quality and type of physical rehabilitation should be considered in future studies. In addition, it is necessary to link injuries with objective load indicators, maturation status, including PHV and post PHV, and the players’ motor profile, to identify potential risk factors for injury in this age group.

## Figures and Tables

**Table 1:** Injury incidence (per 1000 h).

Season	All	Training	Match	IRR 1st–6th Season (95% CI)
**16/17**	7.22 (7.16–7.28)	5.58 (5.53–5.64)	27.87 (27.45–28.29)	0.77 (0.76–0.78)
**17/18**	6.21 (6.16–6.26)	5.41 (5.36–5.46)	16.39 (16.07–16.72)	
**18/19**	7.58 (7.52–7.64)	7.39 (7.33–7.45)	9.84 (9.59–10.09)	
**19/20**	4.49 (4.45–4.55)	3.54 (3.49–3.58)	15.52 (15.20–15.84)	
**20/21**	6.76 (6.71–6.82)	5.34 (5.29–5.40)	24.19 (23.08–24.58)	
**21/22**	9.31 (9.24–9.38)	7.53 (7.46–7.59)	29.51 (29.08–29.94)	
**ALL**	6.95 (6.92–6.97)	5.82(5.79–5.84)	20.6 (20.46–20.75)	

**Table 2:** Seasonal distribution of injured players and total injuries.

Season	Registered Players	Injured Players	Proportion of Injured Players	Total Injuries
**16/17**	46	35	76%	60
**17/18**	48	31	65%	52
**18/19**	48	35	73%	61
**19/20**	52	25	48%	32
**20/21**	42	31	74%	56
**21/22**	53	35	66%	70
	**Total injuries**			331

**Table 3:** Descriptive data of injuries by injury type.

Injury Type	Number of Injuries	Days Out	Reinjury (n)	Training (n)	Match (n)	Non-Contact (n)
**Contusion**	62	8.5	6	39	23	8
**Muscle cramps (DOMS)**	62	12.3	10	42	20	57
**Tendinosis**	49	48.9	8	46	3	45
**Ligament injury (sprain/dislocation)**	57	25.73	13	43	14	32
**Muscle rupture**	41	22.9	6	34	7	31
**Other bone injury**	26	15.2	3	25	1	21
**Fracture**	12	63.6	3	10	2	5
**Lesion of the meniscus or cartilage**	11	109.4	2	10	1	6
**Bursitis**	4	11.8	0	3	1	3
**Tendon rupture**	3	63.7	0	2	1	2
**Concussion**	2	9.5	1	0	2	0
**Abrasion**	1	13	0	1	0	0
**Laceration**	1	1	0	1	0	0

## Data Availability

The data are available upon reasonable request.
